# Vinylbenzyl Chloride/Styrene-Grafted SBS Copolymers via TEMPO-Mediated Polymerization for the Fabrication of Anion Exchange Membranes for Water Electrolysis

**DOI:** 10.3390/polym15081826

**Published:** 2023-04-08

**Authors:** Andrea Roggi, Elisa Guazzelli, Claudio Resta, Gabriele Agonigi, Antonio Filpi, Elisa Martinelli

**Affiliations:** 1 Department of Chemistry and Industrial Chemistry, University of Pisa, Via Moruzzi 13, 56126 Pisa, Italy; 2 Enapter s.r.l., Crespina-Lorenzana (Pisa), 56040 Pisa, Italy

**Keywords:** anion exchange membranes, anion exchange membrane water electrolysis, nitroxide-mediated polymerization, grafting from, green hydrogen, SBS

## Abstract

In this work, a commercial SBS was functionalized with the 2,2,6,6-tetramethylpiperidin-*N*-oxyl stable radical (TEMPO) via free-radical activation initiated with benzoyl peroxide (BPO). The obtained macroinitiator was used to graft both vinylbenzyl chloride (VBC) and styrene/VBC random copolymer chains from SBS to create g-VBC-x and g-VBC-x-*co*-Sty-z graft copolymers, respectively. The controlled nature of the polymerization as well as the use of a solvent allowed us to reduce the extent of the formation of the unwanted, non-grafted (co)polymer, thereby facilitating the graft copolymer’s purification. The obtained graft copolymers were used to prepare films via solution casting using chloroform. The –CH_2_Cl functional groups of the VBC grafts were then quantitatively converted to –CH_2_(CH_3_)_3_N^+^ quaternary ammonium groups via reaction with trimethylamine directly on the films, and the films, therefore, were investigated as anion exchange membranes (AEMs) for potential application in a water electrolyzer (WE). The membranes were extensively characterized to assess their thermal, mechanical, and ex situ electrochemical properties. They generally presented ionic conductivity comparable to or higher than that of a commercial benchmark as well as higher water uptake and hydrogen permeability. Interestingly, the styrene/VBC-grafted copolymer was found to be more mechanically resistant than the corresponding graft copolymer not containing the styrene component. For this reason, the copolymer g-VBC-5-*co*-Sty-16-Q with the best balance of mechanical, water uptake, and electrochemical properties was selected for a single-cell test in an AEM-WE.

## 1. Introduction

Hydrogen is a promising energy vector that has the potential to decarbonize several applications, both in the industrial and domestic fields. However, this goal can only be achieved if hydrogen is produced via water electrolysis, exploiting the excess of energy coming from renewable and less environmentally impacting sources of energy, including wind, hydro, and solar energies [1,2]. At present, most of the globally produced hydrogen is obtained from fossil fuels, and water electrolysis accounts for only a small amount of the total (less than 4%) because of the high production cost. Although the most advanced alkaline water electrolysis benefits from using inexpensive non-noble electrode materials (e.g., nickel and steel), it is, however, affected by large scale production issues, especially at low production rates, and it cannot work with the discontinuous electricity provided by renewable energy sources [3,4]. 

Proton exchange membrane water electrolysis (PEMWE) could overcome some of the drawbacks of alkaline water electrolysis (AWE) technology. In PEMWE, an acidic membrane (Nafion^®^) is used as a solid electrolyte instead of a caustic liquid electrolyte, and the advantages of this technology include high efficiency, a compact design, and high operating current densities, as well as the possibility to be coupled with discontinuous energy sources and to use high pressure on the cathode side, while the anode can operate at atmospheric pressure. On the other hand, the harsh acidic environment of PEMWE limits the choice of suitable catalyst materials to noble metals (Pt), and corrosion-resistant materials are also required for other cell components, including current collectors and bipolar plates. The high costs of such materials as well as of Nafion^®^ are, therefore, another important drawback [2,5].

Anion exchange membrane water electrolysis (AEM-WE) now appears as the most promising technology for hydrogen production, combining the positive aspects of both AWE and PEM-WE. In particular, AEM-WE merges the use of cheaper materials for the fabrication of cell components (e.g., bipolar plates and electrocatalysts) as in AWE and the high-performing working conditions of the solid-state electrolyte-based technologies in PEM-WE, which include higher current density, the possibility to work with a discontinuous electrical supply and with a diluted supporting electrolyte solution or pure water, as well as a compact cell design [6,7]. However, AEM-WE’s early-stage development limits such technology to small-scale production, and the design and preparation of cost-effective, high-performing, and thermally, chemically, and mechanically robust AEMs with high durability under operating conditions still represents one of the main goals for its industrialization [3]. A widespread and straightforward strategy for preparing polymers for AEM production relies on the modification and functionalization of commercially available (co)polymers, including polyolefins [8,9,10], polystyrene [11,12], polysulfone [13,14], polybenzoimidazole [15,16], and polyphenyleneoxide [17,18,19]. However, in order to confer the ability to effectively exchange ions, AEM synthesis requires the introduction of a cationic moiety (more commonly, a quaternary ammonium salt) that in the case of aryl ether repeating units, often results in reduced chemical stability in an alkaline environment due to its electron-withdrawing properties. Even though this aspect can be limited by introducing an alkyl spacer between the polymeric backbone and the positive charges [20,21,22], choosing an aryl-ether-free material is preferred to prevent its potential chemical instability in alkaline conditions from occurring at all. One of the most investigated and currently commercialized AEMs is composed of low-density polyethylene, which is commonly functionalized with vinylbenzyl chloride (VBC) via X-, β-, or γ-ray-induced grafting and then converted to the corresponding ammonium salt with trimethylamine (TMA) [23,24]. Although cheaper than Nafion, polyethylene-based membranes have high costs on the market and suffer from safety concerns and legislative restrictions associated with the grafting process. Therefore, innovation is required to fill this gap. Examples of aryl-ether-free AEMs that have been reported are based on polyolefins or fluorinated polyolefins that were synthesized *ad hoc* starting from the relative monomers [25,26,27,28,29]. On the other hand, an interesting aryl-ether-free commercially available starting material currently under investigation is the poly(styrene-*b*-(ethylene-*co*-butylene)-*b*-styrene) (SEBS) triblock copolymer, a relatively cheap thermoplastic elastomer. This copolymer can be easily functionalized via chloromethylation or acylation to obtain high-performing AEMs [10,30,31]. A similar material is the poly(styrene-*b*-butadiene-*b*-styrene) triblock copolymer (SBS). Different from SEBS, it can be easily functionalized to obtain AEMs by exploiting the reactivity of the double bond in the butadiene repeating unit. Different types of SBS-based AEMs, with excellent electrochemical properties, have been thus prepared, taking advantage of various specific synthetic pathways [32,33]. Among these, the radical polymerization approach seems to be the most interesting one, especially in view of future large-scale production. In this regard, the synthesis of a VBC-grafted SBS copolymer using in-bulk polymerization has been reported. This easy synthetic strategy was effective for preparing AEMs characterized with good electrochemical properties, but the purification of the graft copolymer from the free, i.e., non-grafted, poly(vinylbenzyl chloride) (PVBC) by-product was complicated and required time- and solvent-consuming hot extraction methods [34]. Moreover, the rubbery nature of both SEBS and SBS elastomers, along with the relatively high ionic conductivity, which often results in high water swelling, determine the poor mechanical properties of a membrane under the real operating conditions of AEM-WE. For this reason, further material modifications are usually needed to increase the mechanical robustness, including crosslinking and/or the addition of more rigid components to the polymeric matrix [35,36,37,38].

Starting from this rationale, in this work, we propose the synthesis of graft copolymers via nitroxide-mediated polymerization (NMP) using a 2,2,6,6-tetramethylpiperidin-1-oxyl (TEMPO)-modified SBS thermoplastic elastomer. NMP is, in fact, already reported as being used for the synthesis of copolymers for AEMs [39]. In particular, the vinylbenzyl chloride monomer (VBC) alone or blended with styrene was grafted from the TEMPO-SBS macroinitiator to obtain two sets of graft copolymers, g-VBC-x and g-VBC-x-*co*-Sty-z, respectively. Both sets of graft copolymers were then used for the preparation of AEMs via the amination reaction of the –CH_2_Cl groups with trimethylamine (TMA), and their thermal and mechanical properties as well as water uptake (WU), ion exchange capacity (IEC), in-plane ionic conductivity (*σ*_IP_), and hydrogen permeability (H_2_ P) were determined. 

## 2. Materials and Methods

### 2.1. Materials

Anhydrous toluene was obtained using an mBraun MB SPS5 solvent purifier. HPLC-chloroform (ethanol-stabilized; Carlo Erba, Cornaredo (MI), Italy), chloroform (Carlo Erba, Cornaredo (MI), Italy), methanol (Carlo Erba, Cornaredo (MI), Italy), acetone (Sigma-Aldrich, Darmstadt, Germany), and deuterated solvents (Sigma-Aldrich, Darmstadt, Germany) were used without further purification. 

Vinylbenzyl chloride (VBC) (97% purity; a mixture of 2, 3-, and 4-isomers) and styrene (98% purity) were purchased from Sigma Aldrich (Darmstadt, Germany) and purified via repeating washings with 5% NaOH solutions and water to remove inhibitors. After drying over Na_2_SO_4_, they were distilled under reduced pressure. Benzoyl peroxide (BPO) was purchased from Carlo-Erba (Cornaredo (MI), Italy) and recrystallized from methanol. The 2,2,6,6-Tetramethylpiperidine 1-oxyl (TEMPO) radical was purchased from Sigma-Aldrich (Darmstadt, Germany) (98% purity) and utilized without further purification. Hydroquinone monomethyl ether was purchased from Sigma-Aldrich (Darmstadt, Germany) and utilized without further purification. The white solid was weighed and then dissolved in chloroform to obtain a 125 mg mL^−1^ solution that was stored at 4 °C. Trimethylamine aqueous solution (TMA, 45% wt/v) was purchased from Sigma-Aldrich (Darmstadt, Germany) and used as received.

The poly(styrene-*b*-butadiene-*b*-styrene) (SBS) linear triblock copolymer (SOL T 6302) was purchased from Versalis (Eni, San Donato Milanese (MI), Italy). The polymer was composed of 80 mol% of butadiene (70 mol% of 1,4 and 10 mol% of 1,2 isomers, respectively) with a number average molecular weight of 119,500 g mol^−1^ and a dispersity *Ð* of 1.08. The commercial copolymer was purified via two precipitations in methanol from chloroform solutions.

### 2.2. Synthesis of Graft Copolymers

#### 2.2.1. Synthesis of TEMPO-Grafted SBS Macroinitiator (MI)

The SBS copolymer (7 g, 0.059 mmol) and TEMPO (3.5 g, 22.4 mmol) were added to a 250 mL three-neck round-bottom flask equipped with a magnetic stirrer and condenser. The oxygen was removed via nitrogen/vacuum cycles, and the reactants were dissolved in toluene (65 mL) at room temperature. The reaction mixture was purged with nitrogen for 10 min and heated to 95 °C. Then, a previously degassed solution of BPO (2.22 g, 9.2 mmol in 14 mL of toluene) was added dropwise. The reaction was carried out for 6 h under an inert atmosphere. The product was purified via repeated precipitations into methanol from chloroform solutions. A light brown product (MI) was recovered and dried under vacuum at room temperature (*M*_n_ = 120,000 g mol^−1^; *Ð* = 1.10).

^1^H NMR (CDCl_3_, δ in ppm): 7.2–6.3 (aromatic protons of styrene), 5.6 (CH=CH_2_, 1,2 butadiene), 5.4 ppm (CH=CH, 1,4 butadiene), 5.0 ppm (CH_2_=CH, 1,2 butadiene), 4.1 (HCONR_2_ and H_2_C-ONR_2_), and 2.5–1.2 (aliphatic protons).

#### 2.2.2. Synthesis of g-VBC-x Copolymers

In a typical procedure, the TEMPO-grafted SBS macroinitiator MI (1.75 g, 0,015 mmol) was added into a Carius tube equipped with a magnetic stirrer, and the oxygen was removed via nitrogen/vacuum cycles. Then, it was dissolved in toluene (10.3 mL) at room temperature, and VBC (1.85 mL, 12.8 mmol) was added to the stirred solution. The reaction was carried out at 135 °C for 6 h. The crude product was purified via precipitation into a mixture of acetone/methanol (9/1 *v*/*v*). The brown precipitate was further purified via repeated precipitations into methanol from chloroform solutions. The purified graft copolymer, containing 7 mol% of VBC, was named g-VBC-7.

^1^H NMR (CDCl_3_, δ in ppm): 7.2–6.3 (aromatic protons of styrene and VBC), 5.6 (CH=CH_2_, 1,2 but), 5.4 ppm (CH=CH, 1,4 but), 5.0 ppm (CH_2_=CH, 1,2 but), 4.6–4.3 (CH_2_Cl), 4.1 (HCONR_2_ and H_2_CONR_2_), and 2.5–1.2 (aliphatic protons).

#### 2.2.3. Synthesis of g-VBC-x-*co*-Sty-z Copolymers

In a typical procedure, the TEMPO-grafted SBS macroinitiator MI (2.3 g, 0.019 mmol) was added into a Carius tube equipped with a magnetic stirrer, and the oxygen was removed via nitrogen/vacuum cycles. Then, it was dissolved in toluene (14.5 mL) at room temperature, and VBC (1.5 mL, 10.6 mmol) and styrene (5 mL, 43.7 mmol) monomers were added to the stirred solution. The reaction was carried out at 135 °C for 6 h. The crude product was purified via precipitation in a mixture of acetone/methanol (9/1 *v*/*v*). The brown precipitate was further purified via repeated precipitations into methanol from chloroform solutions. The purified graft copolymer containing 5 mol% of VBC and 16 mol% of styrene was named g-VBC-5-*co*-Sty-16.

### 2.3. Preparation of the Anion Exchange Membranes

#### 2.3.1. Preparation of Polymeric Films

In a typical procedure, a grafted copolymer (680 mg) was dissolved in chloroform at room temperature. The solution was then filtered through filter paper, and a few drops of a chloroform solution of hydroquinone monomethyl ether (0.38 mL of a 125 mg L^−1^ solution) were added under stirring. The solution was sonicated for 5 min and then poured into a PTFE (polytetrafluoroethylene) Petri dish. The Petri dish was left overnight under the fume hood covered with a beaker to slowly evaporate the solvent in a chloroform-saturated atmosphere.

#### 2.3.2. Amination of the Polymeric Films

An aliquot of the aqueous solution of trimethylamine (TMA) (6.5 mL, 45% wt/v) was added to an Erlenmeyer flask containing methanol to obtain a final 2.5% wt/v TMA solution. Grafted copolymer films were immersed in the solution and left for 40 h at 40 °C. The films were then washed repeatedly with methanol and water and dried under vacuum at room temperature. 

### 2.4. Characterization

FT-IR spectra were recorded with a Perkin-Elmer Spectrum One spectrometer (Waltham, MA, USA). Free-standing films were analyzed using FTIR in transmission mode.

^1^H-NMR spectra were recorded with a Bruker Avance 400 DRX (400 MHz, Billerica, MA, USA) spectrometer. Samples were prepared by dissolving 25–30 mg of the product in around 0.8 mL of deuterated chloroform. 

The number and weight average molecular weights were determined using gel permeation chromatography with a Jasco PU-2089Plus liquid chromatograph (Hachioji-shi, Tokyo, Japan) equipped with two PL gel 5 µm mixed-D columns, a Jasco RI-2031Plus refractive index detector (Hachioji-shi, Tokyo, Japan), and a Jasco UV-2077Plus UV/vis detector (Hachioji-shi, Tokyo, Japan). Polystyrene standards were used for calibration (400–400,000 g mol^−1^). Samples were filtered with a 0.2 μm PTFE filter before injection.

Differential scanning calorimetry (DSC) analysis was performed with a TA Instruments Discovery DSC 250 calorimeter (Columbus, OH, USA). Samples weighing between 5 and 10 mg were dried under vacuum at room temperature and stored in a dry environment until the measurement. The glass transition temperature was determined as the inflection point of the curve.

Thermogravimetric analysis was performed using a thermogravimetric analyzer Mettler TGA Q500 (Columbus, OH, USA). Samples of approximately 5 mg in weight were prepared and analyzed under a nitrogen inert flux of 80 mL min^−1^ via heating from 30 °C to 700 °C at 10 °C min^−1^.

#### 2.4.1. Characterization of the Anion Exchange Membranes

The mechanical properties of the membranes were evaluated using stress–strain tests. Dogbone-shaped specimens (21.1 mm × 4.75 mm) were cut and conditioned overnight in a 1 M NaHCO_3_ solution at room temperature. The specimens were swabbed with filter paper. The stress–strain properties at a grip separation rate of 211 mm min^−1^ of the samples were recorded with a Tinius Olsen instrument.

Water uptake (WU) was determined according to the following procedure. The dry weight (*W*_dry_) of a membrane in Cl^−^ form was measured after drying overnight under vacuum at room temperature. The membrane was then conditioned in a 1 M KOH aqueous solution for approximately 12 h at room temperature. The wet weight (*W*_KOH_) was measured after rinsing the sample with deionized water to remove the excess adsorbed KOH and was wiped with humidified filter paper. The water uptake was calculated using Equation (1):

(1) $$WU_{\mathrm{KOH}}\left(\%\right)=\frac{W_{\mathrm{KOH}}-W_{\mathrm{Dry}}}{W_{\mathrm{Dry}}}\cdot 100$$

In-plane ionic conductivity (*σ_IP_*) measurements were performed with a multi-channel potentiostat/galvanostat with an impedance channel (VMP3 from Bio-Logic SA, Seyssinet-Pariset, France) for electrochemical impedance spectroscopy (EIS) measurements. Samples were cut and conditioned for a night at room temperature in a 1 M KHCO_3_ aqueous solution. Afterward, the samples were washed with deionized water and put in a BekkTech PTFE 4-probe flow-cell assembled with a 5 cm^2^ cell hardware provided by Fuel Cell Technologies inc. The excess electrolyte was removed by injecting a continuous flow of N_2_-degassed water before the measurements. Degassed liquid water was fed for the entire duration of the test. Resistance values were obtained by fitting the impedance data to an RC circuit between 100 kHz and 10 kHz (i.e., before the low-frequency arc) to prevent artifacts. The membrane resistivity was calculated from the resistance values according to Equation (2):

(2) $$\rho \left(\Omega \;\mathrm{cm}\right)=\frac{HFR\;\left(\Omega \right)\cdot A\left({\mathrm{cm}}^{2}\right)}{L\;\left(\mathrm{cm}\right)}$$

where HFR is the measured resistance from impedance spectra, *A* is the area (obtained by multiplying the Pt wires distance and membrane width), and L is the sample thickness. The ionic conductivity was then calculated according to Equation (3):

(3) $$\sigma_{\mathrm{IP}}\left(m\;S/\mathrm{cm}\right)=\frac{1}{\rho \left(\Omega \;\mathrm{cm}\right)}\cdot 1000$$

The ion exchange capacity (IEC) of a membrane was measured using acid–base back titration. Specifically, the membrane was previously cut into pieces and dried under vacuum at room temperature for 24 h. The dried strips were weighed in a closed vessel and then immersed in 1 M KOH and left overnight at room temperature. Then, the pieces were washed repeatedly with water and soaked for 3 h in a measured volume of a standardized aqueous HCl solution, whereby the OH^−^ in the membrane was neutralized by the acid. After 3 h at room temperature, the membrane pieces were washed with deionized water. The excess HCl was then titrated by using a standardized NaOH solution. The end-point was detected using the conductometric method. The IEC was calculated according to Equation (4):

(4) $$IEC\;\left(\frac{meq}{g}\right)=\frac{meq_{\mathrm{HCl}}-meq_{\mathrm{NaOH}}}{g_{\mathrm{polymer}}}$$

where *meq*_HCl_ and *meq*_NaOH_ represent the milliequivalent of the HCl solution used for soaking the membrane during the neutralization of its OH^−^ groups and the milliequivalent of the NaOH solution used for the titration of the excess acid, respectively; *g*_polymer_ is the weight of the dry membrane. 

Hydrogen permeability through the membrane was determined with electrochemical measurements. A membrane electrode assembly (MEA) was assembled by placing the membrane, previously conditioned in 1 M KOH, between 2 electrodes containing approximately 0.2 mg cm^−2^ of platinum using a commercial Pt/C catalyst. The MEA was then placed in a 5 cm^2^ cell hardware provided by Fuel Cell Technologies inc. Then, the cell was connected to a test station for hydrogen fuel cells, which was used to control the gas flow at the anode and cathode and their relative humidity (RH). The system was conditioned by flowing fully humidified H_2_ (99.999% Nippon gases) and N_2_ (99.999% Nippon gases) at the cathodic and anodic sides, respectively, until the measured open circuit electric potential reached 0.3 V or less. Then, the cell was equilibrated by performing cyclic voltammetry ranging from 0.05 to 0.8 V with a sweep rate of 60 mV s^−1^ until reproducible and characteristic voltammograms were recorded. Finally, chronoamperometry at 0.35 V was performed until a stable current value was reached. Hydrogen permeability through a membrane correlates to the measured current according to Faraday’s law [40].

#### 2.4.2. Electrolytic Cell Tests

AEM-WE tests were carried out using Enapter’s cell hardware and electrodes with a dry cathode patented configuration at an operating current density of 800 mA/cm^2^, 55 °C, and a cathodic pressure of 30 bar. A 1 wt% KOH solution was fed as a supporting electrolyte.

The single-cell performances were evaluated using battery test equipment from Arbin Instruments (LBT 5V-10A, College Station, TX, USA)

## 3. Results and Discussion

### 3.1. Synthesis of the Graft Copolymers

**Figure 1:**
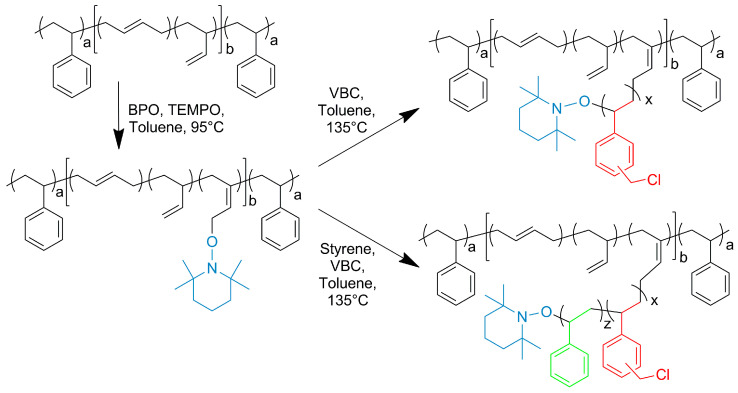
Synthesis of the grafted copolymers g-VBC-x and g-VBC-x-*co*-Sty-z and the corresponding TEMPO-based SBS macroinintiator. a and b are the mole percentages of styrene and butadiene units in the SBS block copolymer, respectively. x and z are the mole percentages of VBC and styrene units in the grafted chains, respectively. The blue, red and green colors indicate the grafted units of TEMPO, VBC and styrene, respectively on the SBS backbone.

**Figure 2:**
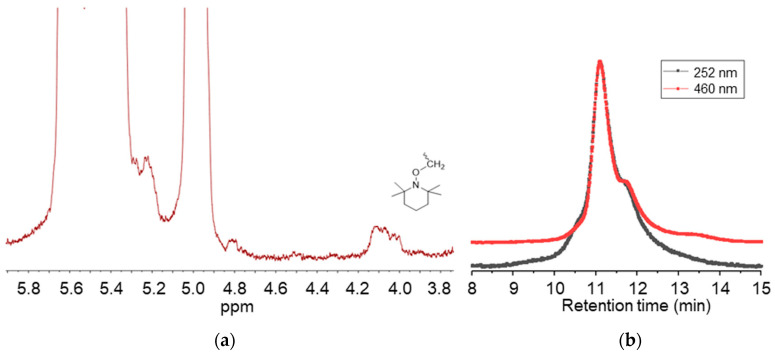
^1^H-NMR spectrum in the region 3.8–5.9 ppm of the macroinitiator MI (**a**), and GPC curves of the macroinitiator MI obtained with the UV detector at λ = 252 nm and λ = 460 nm (**b**).

g-VBC-x and g-VBC-x-*co*-Sty-z were synthesized via a grafting from reaction starting from a TEMPO-functionalized SBS macroinitiator. The functionalization of the SBS copolymer with TEMPO was conducted in a toluene solution using BPO as a radical initiator (Figure 1). Primary radicals generated from the BPO initiator have been reported to react most likely via the hydrogen abstraction of allylic hydrogen on 1,2-butadiene units, thus creating allylic macroradicals to which the TEMPO units are anchored [34]. The ^1^H NMR analysis of TEMPO-functionalized SBS (MI) presented a broad signal at 4.1 ppm due to protons of the newly generated alkoxyamine group (Figure 2a) that was used to estimate the mole percentage of TEMPO moieties (0.2%) with respect to butadiene repeating units in the copolymer. The covalent attachment of TEMPO to the SBS polymer backbone was also confirmed with GPC measurements performed with a UV-vis detector set at the wavelength of TEMPO absorbance (λ = 460 nm). The elution curves of the macroinitiator acquired at λ = 460 nm had approximately the same shape and retention time as that obtained at λ = 252 nm (Figure 2b). 

The purified macroinitiator was then used for grafting VBC and a mixture of VBC and styrene to obtain the g-VBC-x and g-VBC-x-*co*-Sty-z copolymers. In both cases, the grafting reaction was carried out in a toluene solution at 135 °C for 8 h (Figure 1). At this temperature, the homolytic cleavage of the C-O bond of the alkoxyamine occurs, and the macroinitiator can start the monomer(s) polymerization. At the end of the polymerization, the crude graft copolymers were purified via 2 precipitation steps, the first, into acetone/methanol 9:1 *v*/*v* in order to remove the free, i.e., non-grafted, PVBC homopolymer or VBC-*co*-Sty copolymer. The second precipitation into methanol allowed the purication of the product from any residual non-reacted monomer. The effective grafting of VBC from the SBS backbone was proven using ^1^H NMR and GPC analysis. In particular, the functionalization degree (FD) (Table 1), which is the mole percentage of VBC in the final copolymer, was calculated from the integrated area of the signal at 4.5 ppm (A_VBC_) due to the CH_2_Cl group of the VBC, according to the Equation (5):

(5) $${\mathrm{FD}}_{\mathrm{VBC}}\;\left(\mathrm{mol}\%\right)=\mathrm{mol}\%\;\mathrm{VBC}=\frac{\frac{A_{\mathrm{VBC}}}{n_{\mathrm{VBC}}}}{\sum_{i}\frac{A_{i}}{n_{i}}}\cdot 100$$

where A_i_ and n_i_ are the integrated area and number of hydrogen atoms, respectively, of each individual chemical group, namely, the aromatic protons of styrene, that were calculated by subtracting the VBC contribution from the total signal between 7.2 and 6.3 ppm, the CH=CH of 1,4 butadiene at 5.4 ppm, the CH=CH_2_ of 1,2 butadiene at 5.0 ppm, and the CH_2_Cl of VBC between 4.6 and 4.3 ppm. 

**Figure 3:**
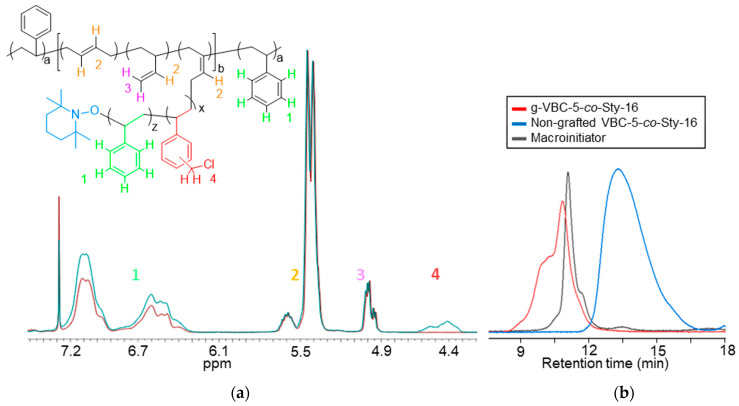
^1^H NMR spectra of the purified g-VBC-5-*co*-Sty-16 copolymer (cyan line) and the starting macroinitiator MI (red line) (**a**). The color of the different protons was chosen as follows: green for styrene, red for VBC, yellow for 1,4-butadiene at 5.6 ppm and 1,2-butadiene at 5.4 ppm, and purple for 1,2-butadiene at 5.0 ppm. GPC curves of the purified g-VBC-5-*co*-Sty-16 copolymer, the starting macroinitiator, and the non-grafted copolymer obtained using the UV-detector at λ = 252 nm (**b**).

The GPC analysis confirmed the absence of free VBC homopolymer in the final copolymer and, therefore, the effectiveness of the purification procedure. The relative amounts of VBC and styrene in the g-VBC-x-*co*-Sty-z copolymers were also evaluated by means of ^1^H NMR, taking into consideration the signals at 4.5 ppm and 7.3–6.3 ppm due to the CH_2_Cl protons of VBC and the aromatic protons of both VBC and the styrene units, respectively. The mole ratios of VBC/Sty in the side chains resulted to be 24/76 and 43/57 for g-VBC-5-*co*-Sty-16 and g-VBC-10-*co*-Sty-13, respectively which was consistent with the fed one (Table 1). The GPC analysis demonstrates that the copolymers had higher molecular weights than the starting SBS macroinitiator (Figure 3b and Table 1) and did not contain the non-grafted polymer, as the blue curve in Figure 3b is well-separated from the red one of the purified graft copolymer. This result indicates that the purification procedure was also very effective for the removal of the free copolymer.

With respect to previous works reported in the literature on the preparation of g-VBC copolymers via in-bulk free-radical polymerization [34,41], the use of a solvent as well as a controlled radical polymerization technique represents a significant step forward, as a smaller amount of free homopolymer or copolymer (<10 wt%) was obtained, and it was generally characterized by a lower molecular weight (*M*_n_ < 15,000 g mol^−1^), thus making the purification process easier without the use of time-, energy- and solvent-consuming hot extraction methods.

### 3.2. Film Preparation

**Figure 4:**
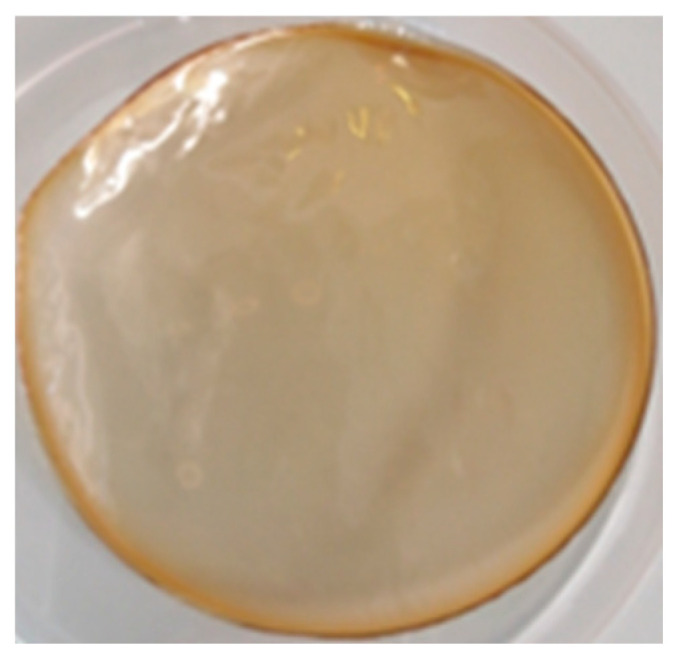
Image of a cast graft copolymer film.

Films were prepared by casting the chloroform solutions of the graft copolymers (~13.5 mg mL^−1^) into PTFE Petri dishes. The solutions also contained 70 ppm of hydroquinone monomethyl ether, as a radical inhibitor, to prevent aging phenomena in the films and improve their stability over time. Before casting, the copolymer solutions were paper-filtered and sonicated. Complete solvent evaporation took approximately two days and was conducted in a CHCl_3_-saturated environment. The obtained films appeared to be free from macroscopic defects and homogeneous in thickness (70–100 μm) (Figure 4). 

**Figure 5:**
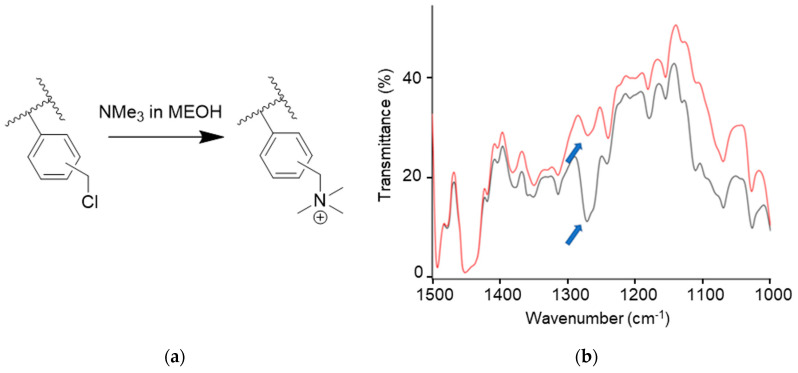
Quaternization reaction of chlorobenzyl groups in the graft copolymers (**a**). FT–IR spectra in the region 1000–1500 cm^−1^ for the films before (black line) and after (red line) quaternization reaction (**b**).

The films were quaternized via immersion in a solution of trimethylamine (TMA) in methanol (2.5% wt/v) at 40 °C for 40 h (Figure 5a). The progress of the reaction was monitored using FT-IR spectroscopy in transmission mode, taking into consideration the disappearance of the peak at around 1265 cm^−1^ that is ascribable to the CH_2_Cl wagging of the chlorobenzyl group (Figure 5b). The films after quaternization were named g-VBC-x-*co*-Sty-z-Q. 

### 3.3. Thermal Characterization

#### 3.3.1. Thermogravimetric Analysis 

The thermal stability of anion exchange membranes plays a crucial role in their application in electrochemical systems. The g-VBC-x and g-VBC-x-*co*-Sty-z grafted copolymers before and after the quaternization reaction were studied using thermal gravimetric analysis (TGA) performed under a nitrogen atmosphere starting from room temperature up to 700 °C with a heating rate of 10 °C min^−1^.

**Figure 6:**
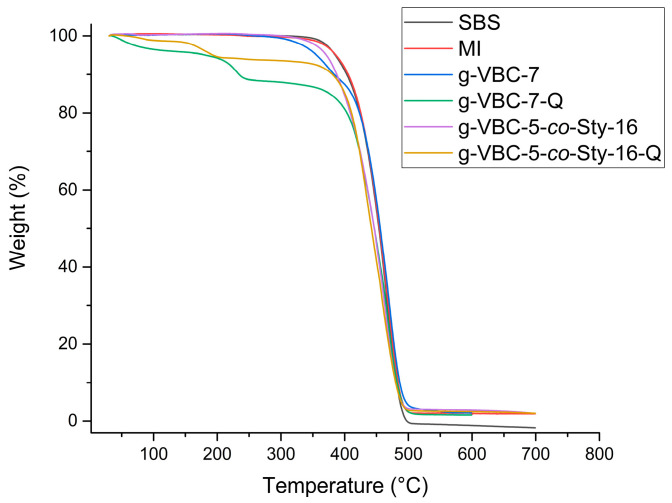
TGA traces of SBS, MI, g-VBC-7, g-VBC-7-Q, g-VBC-5-*co*-Sty-16, and g-VBC-5-*co*-Sty-16-Q.

The synthesized copolymers degraded in a two-step process, different from that of the corresponding TEMPO-based macroinitiator and the pristine SBS, which presented a single weight loss (Figure 6) (Table 2). Moreover, the VBC-grafted copolymers showed a temperature of initial degradation (*T*_onset_^1^) significantly lower (*T*_onset_^1^ = 260–300 °C) than that of the corresponding TEMPO-based macroinitiator (*T*_onset_^1^ = 400–420 °C), which in turn was comparable to that of the unmodified SBS (*T*_onset_^1^ = 420 °C) (Figure 6) (Table 2). The first weight loss was attributed to the degradation of the VBC-grafted chains, consistent with what is reported in the literature [42,43], while the second one was due to the SBS backbone. The weight loss associated with the first step was generally found to correlate with the weight percentage of VBC in the corresponding copolymer. After the quaternization reaction, *T*_onset_ significantly decreased as a result of the conversion of the chlorobenzyl moieties into the more thermally unstable quaternary ammonium groups, for example, decreasing from 366 °C to 149 °C for the g-VBC-5-*co*-Sty-16 film before and after the quaternization reaction, respectively. These results confirm that the quaternized membranes were thermally stable at the operating thermal conditions (*T* ≤ 60 °C) for the considered electrochemical applications.

#### 3.3.2. Differential Scanning Calorimetry

Both SBS and the TEMPO-functionalized SBS macroinitiator displayed similar thermal behavior, characterized by the presence of 2 different glass transition temperatures, *T*_g1_ and *T*_g2_, at ~−90 °C and in the range 83–90 °C, respectively. While the first one was due to the polybutadiene block, the second *T*_g_ was ascribable to the rigid polystyrene blocks (Table 3). Similar thermal behavior was also displayed by the g-VBC-x and g-VBC-x-*co*-Sty-z copolymers, indicating that the styrene and VBC blocks were miscible and provided a single thermal transition. In the literature, styrene-*b*-VBC diblock copolymers were reported to show a single *T*_g_ as a result of the chemical compatibility of the two blocks, at least for some specific copolymer compositions [44]. The results also demonstrate that the polymer films were phase-separated (to a certain extent) in bulk into different domains, whereby one was preferentially composed of polybutadiene, while the other was composed of the compatible blend of VBC and styrene. 

**Figure 7:**
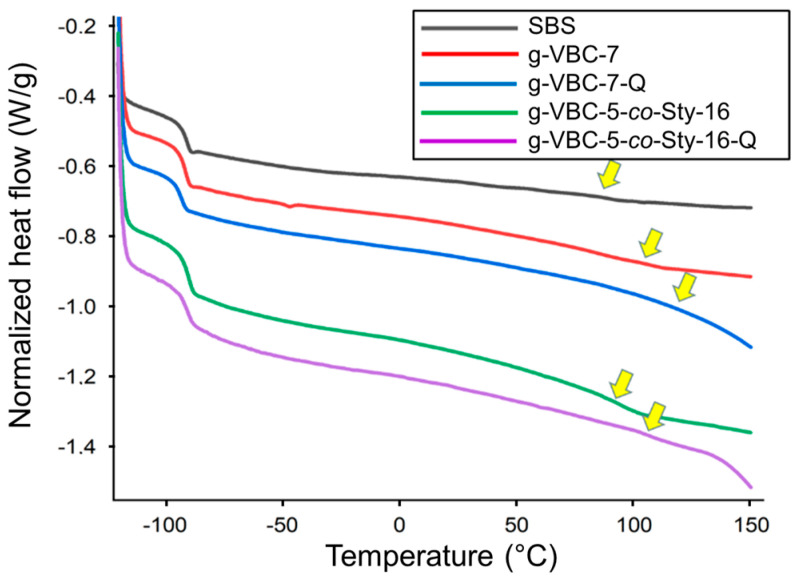
DSC second heating curves (endo down) for SBS, g-VBC-7, g-VBC-7-Q, g-VBC-5-*co*-Sty-16, and g-VBC-5-*co*-Sty-16-Q.

The films after quaternization were still characterized by two distinct glass transition temperatures (Figure 7). In particular, *T*_g1_ was approximately −90 °C, suggesting that the amination process as well as the grafting reaction did not affect the thermal behavior of the polybutadiene block. On the other hand, *T*_g2_ was detected at a temperature higher than that of the respective copolymer before amination (approximately 110 °C), which is consistent with the formation of polar interactions between the charged groups in the film post-amination.

### 3.4. Mechanical Properties

In AEM electrolyzers, a membrane is subjected to mechanical stresses during the assembly of the membrane electrode assembly (MEA) and during working conditions. It is, therefore, important that the membrane have a mechanical resistance and robustness that is suitable for the final application. The mechanical properties of the membranes were investigated using tensile stress–strain tests. These tests were performed after conditioning the dogbone-shaped specimens overnight in a 1 M KHCO_3_ aqueous solution in order to evaluate the membrane properties under conditions as close as possible to the electrolytic cell working conditions. A KHCO_3_ solution was used in place of KOH to avoid carbonation phenomena. Differently, the SBS starting material was evaluated as a dry sample, given its hydrophobic character. The Young’s moduli (*E*), stress ($\sigma_{R}$) values, and deformation at break ($\varepsilon_{R}$) values of the tested films are reported in Table 4.

**Figure 8:**
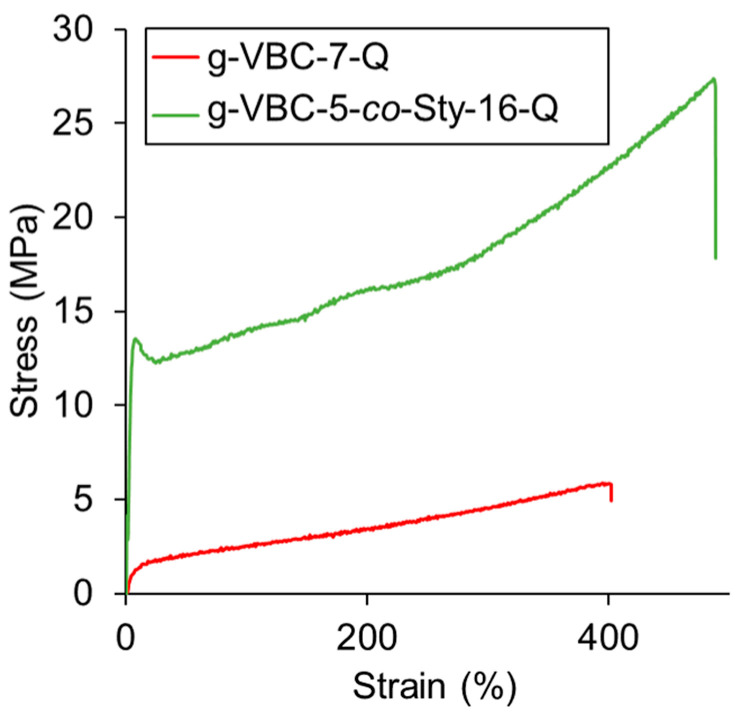
Stress–strain curves for SBS, g-VBC-7-Q, and g-VBC-5-*co*-Sty-16-Q.

As expected, the SBS starting material displayed an elastomeric behavior, being characterized by a low elastic modulus and a high elongation at break. As evident in Table 4, the conditioned g-VBC-7-Q membrane presented a lower elastic modulus than SBS due to the plasticizing effect of water. On the other hand, the inclusion of a rigid and apolar comonomer, such as styrene, in the side chains led to a membrane with an enhanced elastic modulus (Figure 8). 

### 3.5. Water Uptake and Electrochemical Properties

In order to determine the suitability of the graft copolymer membranes for use in AEM-WE, the IEC, WU, *σ*_IP_, and hydrogen permeability of the films were investigated. The values obtained for each parameter were compared with those reported in the literature [27] for a commercially available AEM, the A-201 from Tokuyama Corporation, used as the benchmark. The IEC values of the graft copolymer membranes increased with the increase in the functionalization degree, passing from 0.88 meq g^−1^ for FD = 7% to 1.9 meq g^−1^ for FD = 22%. A similar observation was also valid for the g-VBC-x-*co*-Sty-z-Q membranes, which also displayed lower IEC values with respect to the corresponding g-VBC-x-Q membranes with similar FDs. This was due to the presence in the side chains of styrene, a non-polar and non-ionic conductive comonomer. All the membranes presented an IEC lower than the benchmark, the only exception being g-VBC-22-Q, the membrane with the highest FD. The functionalization degree also affects the other two important parameters, namely, conductivity and water uptake, which are both strictly related to IEC. As shown in Table 5, both ionic conductivity and water uptake increased with the increase in the functionalization degree.

In particular, WU was, in all cases, higher than 100%. Moreover, it was lower for g-VBC-x-*co*-Sty-z-Q membranes than for their g-VBC-x-Q counterparts with comparable FDs, thanks to the inclusion of the apolar, non-swellable styrene component.

A similar trend was also observed for the ionic conductivity values of the membranes, which were, in all cases, comparable to or even higher than that of the benchmark, with the maximum being measured for g-VBC-22-Q, the membrane with the highest FD. However, its high WU caused very marked hydration in the operating conditions, which made it mechanically poor.

Hydrogen permeability is another important parameter to take into consideration in a working electrolytic cell. Low hydrogen permeability through a polymeric membrane from the cathode to the anode is important (i) to prevent safety concerns in operative conditions related to the possible formation of explosive hydrogen/oxygen mixtures and (ii) for the efficiency of the cell, as higher permeability leads to a higher loss in hydrogen production. The values obtained for the copolymer graft membranes were relatively high and correlated to the swelling of the materials, being higher for the membranes with higher WUs. However, the g-VBC-5-*co*-Sty-16-Q membrane presented a value that was slightly higher than the maximum accepted value (9.0·10^−17^ mol cm^−1^s^−1^Pa^−1^).

### 3.6. Electrolysis Test

The g-VBC-5-*co*-Sty-16-Q membrane was selected for an *in operando* cell test in an AEM-WE, having the most balanced mechanical and *ex situ* electrochemical properties. In fact, the attempt at using the g-VBC-7-Q membrane failed during its assembling because of its low mechanical robustness.

**Figure 9:**
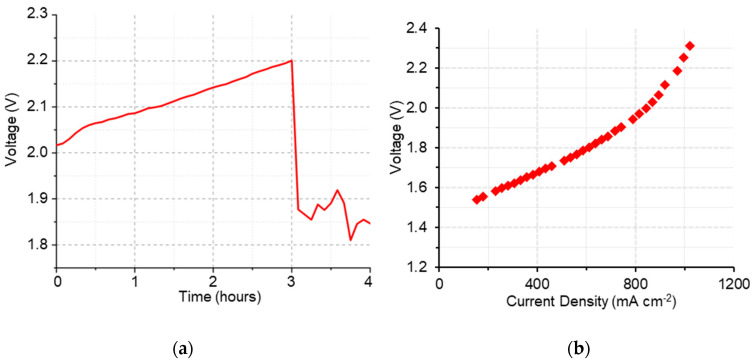
Durability (**a**) and polarization (**b**) curves of g-VBC-5-*co*-Sty-16-Q membrane during electrolysis test.

Although the membrane overcame the first stage of conditioning, once in durability, the potential started from very high values (> 2 V) and significantly increased over the first 3 h (Figure 9a), showing high resistance and mass transport issues, as shown in the IV curve (Figure 9b). This was also confirmed by the relatively high value of cell resistance obtained using the current interrupt method (approximately 0.037 Ω for the entire duration of the test). The poor in situ performance of g-VBC-5-*co*-Sty-16-Q might be associated with its modest ion conductivity (5.7 mS cm^−1^ in HCO_3_^−^ form) and relatively high thickness of ~100 µm that was necessary to guarantee a sufficient mechanical robustness and lower hydrogen permeability in consideration of the relatively high hydrogen permeability (ex situ data).

## 4. Conclusions

TEMPO-mediated polymerization was effective for the preparation of graft copolymers composed of an SBS backbone and VBC or VBC/Sty random copolymer side chains. The controlled nature of the polymerization as well as the use of a solvent for the reaction led to a reduction in the non-grafted polymer and an easier and more effective purification procedure. For a given functionalization degree, the membranes deriving from the graft copolymers containing styrene in the side chains showed improved mechanical properties as well as reduced water uptake and ionic conductivity with respect to the copolymers without styrene.

The test in single electrolysis cell confirmed that mechanical robustness is a key factor that should always be addressed during AEM design and preparation. In fact, the incorporation of significant amounts of styrene in the grafted chains guaranteed the resistance of the membrane during MEA assembly and the conditioning stage, even though the cell voltage at the operating current density of 800 mA/cm^2^ was too high for practical application. Although the membrane cell performance needs to be improved, these results indicate that VBC-functionalized SBS-based copolymers with an increased amount of the styrene component are good candidates to easily prepare cost-effective AEMs for green hydrogen production.

## 5. Patents

A patent was produced from the research work reported in this paper. The invention relates to an anion exchange membrane and a method for manufacturing an AEM, which is under the protection of the World Intellectual Property Office, as an invention patent, with international publication number WO2022090545A1, application number PCT/EP2021/080277, entitled: “Ion exchange membrane and method of manufacturing an ion exchange membrane”.

## Figures and Tables

**Table 1:** Properties of the synthesized copolymers.

Graft Copolymer	FD_VBC_ (%)	VBC ^a^ wt/wt	Sty ^a^ wt/wt	VBC ^b^ (mol%)	VBC ^c^ (mol%)	*M*_n_ ^d^ (g mol^−1^)	*M*_n_ ^e^ (g mol^−1^)	*Ð*
g-VBC-7	7	1.1	-	100	100	137,000	19,500	1.50
g-VBC-22	22	3.6	-	100	100	150,000	63,500	1.60
g-VBC-5-*co*-Sty-16	5	0.7	1.98	20	24	158,000	55,200	1.58
g-VBC-10-*co*-Sty-13	10	1.45	1.52	40	43	201,000	45,600	1.56

^a^ Referred to SBS. ^b^ Mole percentage of VBC in the fed VBC+Sty mixture. ^c^ Mole percentage of VBC in the grafted chains. ^d^ Average number molecular weight of the copolymer using GPC. ^e^ Average number molecular weight of the grafted chains calculated by ^1^H NMR.

**Table 2:** Thermogravimetric data of the grafted copolymers before and after quaternization reaction, the corresponding macroinitiator, and SBS.

Film	*T*_onset_^1 a^ (°C)	*T*_max_^1 b^ (°C)	*T*_onset_^2 c^ (°C)	*T*_max_^2 d^ (°C)	Residue at 700 (%wt)
SBS	420	460	-	-	0
MI	406	468	-	-	2
g-VBC-7	333	367	408	467	2
g-VBC-7-Q ^e^	202	230	408	467	2
g-VBC-5-*co*-Sty-16	346	380	420	457	2
g-VBC-5-*co*-Sty-16-Q ^e^	149	164	404	454	4

**^a^** Initial thermal degradation temperature of the first degradation step. **^b^** Temperature of the maximum weight loss rate of the first degradation step. **^c^** Initial thermal degradation temperature of the second degradation step. ^d^ Temperature of the maximum weight loss rate of the second degradation step. ^e^ Films after quaternization reaction.

**Table 3:** DSC data of SBS, MI, and graft copolymers.

Film	*T*_g1_ ^a^ (°C)	ΔCp_1_^b^ (J (g K)^−1^)	*T*_g2_^a^ (°C) ^a^	ΔCp_2_^b^ (J (g K)^−1^)
SBS	−91	0.47	90	0.05
MI	−89	0.45	90	0.11
g-VBC-7	−91	0.40	81	0.30
g-VBC-7-Q ^c^	−93	0.25	115	0.18
g-VBC-5-*co*-Sty-16	−90	0.35	95	0.26
g-VBC-5-*co*-Sty-16-Q^c^	−90	0.29	108	0.10

^a^ *T*_g1_ and *T*_g2_ values from the second heating cycle. ^b^ Heat capacity variation corresponding to glass transition temperature. ^c^ Films after quaternization reaction.

**Table 4:** Mechanical properties of the aminated membranes and the SBS starting material.

Film	*E* (MPa) ^a^	$\varepsilon_{R}$ (%)	$\sigma_{R}$ (MPa)
SBS	30 ± 10	670 ± 90	6 ± 3
g-VBC-7-Q ^b^	22 ± 1	370 ± 50	6 ± 1
g-VBC-5-*co*-Sty-16-Q ^b^	260 ± 30	460 ± 30	25 ± 2

^a^ Elastic modulus determined as the slope of the linear range of the stress–strain curve. ^b^ Films after quaternization reaction.

**Table 5:** Electrochemical properties of the aminated membranes.

Film	*σ*_IP_ ^a^ (mS cm^−1^)	H_2_ P (mol cm^−1^s^−1^Pa^−1^)	IEC (meq g^−1^)	WU (%)
Benchmark ^b^	7.3	-	1.84 ± 0.04	44 ± 5
g-VBC-7-Q	6.4	17.7·10^−17^	0.88 ± 0.01	130 ± 30
g-VBC-22-Q	19.8	n.d. ^c^	1.9 ± 0.2	510 ± 40
g-VBC-5-*co*-Sty-16-Q	5.7	11.9·10^−17^	0.6 ± 0.1	110 ± 30
g-VBC-10-*co*-Sty-13-Q	7.6	18.4·10^−17^	1.5 ± 0.3	200 ± 50

^a^ In-plane ion conductivity determined at 25 °C in HCO_3_^−^ form. ^b^ Literature data reported in [27]. ^c^ Not determined.

## References

[1] Chi J., Yu H. (2018). Water electrolysis based on renewable energy for hydrogen production. Chin. J. Catal..

[2] Vincent I., Bessarabov D. (2018). Low cost hydrogen production by anion exchange membrane electrolysis: A review. Renew. Sustain. Energy Rev..

[3] Henkensmeier D., Najibah M., Harms C., Žitka J., Hnát J., Bouzek K. (2021). Overview: State-of-the Art Commercial Membranes for Anion Exchange Membrane Water Electrolysis. J. Electrochem. Energy Convers. Storage.

[4] Miller H.A., Bouzek K., Hnat J., Loos S., Bernäcker C.I., Weißgärber T., Röntzsch L., Meier-Haack J. (2020). Green hydrogen from anion exchange membrane water electrolysis: A review of recent developments in critical materials and operating conditions. Sustain. Energy Fuels.

[5] Khataee A., Shirole A., Jannasch P., Krüger A., Cornell A. (2022). Anion exchange membrane water electrolysis using Aemion™ membranes and nickel electrodes. J. Mater. Chem. A.

[6] Yang Y., Li P., Zheng X., Sun W., Dou S.X., Ma T., Pan H. (2022). Anion-exchange membrane water electrolyzers and fuel cells. Chem. Soc. Rev..

[7] Park E.J., Arges C.G., Xu H., Kim Y.S. (2022). Membrane Strategies for Water Electrolysis. ACS Energy Lett..

[8] Zhang M., Shan C., Liu L., Liao J., Chen Q., Zhu M., Wang Y., An L., Li N. (2016). Facilitating Anion Transport in Polyolefin-Based Anion Exchange Membranes via Bulky Side Chains. ACS Appl. Mater. Interfaces.

[9] Zhu M., Su Y., Wu Y., Zhang M., Wang Y., Chen Q., Li N. (2017). Synthesis and properties of quaternized polyolefins with bulky poly(4-phenyl-1-butene) moieties as anion exchange membranes. J. Membr. Sci..

[10] Jeon J.Y., Tian D., Pagels M.K., Bae C. (2019). Efficient Preparation of Styrene Block Copolymer Anion Exchange Membranes via One-Step Friedel–Crafts Bromoalkylation with Alkenes. Org. Process. Res. Dev..

[11] Liu W., Liu L., Liao J., Wang L., Li N. (2017). Self-crosslinking of comb-shaped polystyrene anion exchange membranes for alkaline fuel cell application. J. Membr. Sci..

[12] Xue J., Liu L., Liao J., Shen Y., Li N. (2017). UV-crosslinking of polystyrene anion exchange membranes by azidated macromolecular crosslinker for alkaline fuel cells. J. Membr. Sci..

[13] Chen W., Hu M., Wang H., Wu X., Gong X., Yan X., Zhen D., He G. (2017). Dimensionally stable hexamethylenetetramine functionalized polysulfone anion exchange membranes. J. Mater. Chem. A.

[14] Weiber E.A., Jannasch P. (2015). Polysulfones with highly localized imidazolium groups for anion exchange membranes. J. Membr. Sci..

[15] Hao J., Jiang Y., Gao X., Lu W., Xiao Y., Shao Z., Yi B. (2018). Functionalization of polybenzimidazole-crosslinked poly(vinylbenzyl chloride) with two cyclic quaternary ammonium cations for anion exchange membranes. J. Membr. Sci..

[16] Li S., Zhu X., Liu D., Sun F. (2018). A highly durable long side-chain polybenzimidazole anion exchange membrane for AEMFC. J. Membr. Sci..

[17] Zhu L., Yu X., Hickner M.A. (2018). Exploring backbone-cation alkyl spacers for multi-cation side chain anion exchange membranes. J. Power Sources.

[18] Yang Y., Knauss D.M. (2015). Poly(2,6-dimethyl-1,4-phenylene oxide)-*b*-poly(vinylbenzyltrimethylammonium) Diblock Copolymers for Highly Conductive Anion Exchange Membranes. Macromolecules.

[19] Pan J., Han J., Zhu L., Hickner M.A. (2017). Cationic Side-Chain Attachment to Poly(Phenylene Oxide) Backbones for Chemically Stable and Conductive Anion Exchange Membranes. Chem. Mater..

[20] He Y., Ge X., Liang X., Zhang J., Shehzad M.A., Zhu Y., Yang Z., Wu L., Xu T. (2018). Anion exchange membranes with branched ionic clusters for fuel cells. J. Mater. Chem. A.

[21] Han J., Pan J., Chen C., Wei L., Wang Y., Pan Q., Zhao N., Xie B., Xiao L., Lu J. (2019). Effect of Micromorphology on Alkaline Polymer Electrolyte Stability. ACS Appl. Mater. Interfaces.

[22] Liu L., Chu X., Liao J., Huang Y., Li Y., Ge Z., Hickner M.A., Li N. (2018). Tuning the properties of poly(2,6-dimethyl-1,4-phenylene oxide) anion exchange membranes and their performance in H_2_/O_2_ fuel cells. Energy Environ. Sci..

[23] Douglin J.C., Varcoe J.R., Dekel D.R. (2020). A high-temperature anion-exchange membrane fuel cell. J. Power Sources Adv..

[24] Meek K.M., Reed C.M., Pivovar B., Kreuer K.-D., Varcoe J.R., Bance-Soualhi R. (2020). The alkali degradation of LDPE-based radiation-grafted anion-exchange membranes studied using different ex situ methods. RSC Adv..

[25] Zhang X., Chu X., Zhang M., Zhu M., Huang Y., Wang Y., Liu L., Li N. (2019). Molecularly designed, solvent processable tetraalkylammonium-functionalized fluoropolyolefin for durable anion exchange membrane fuel cells. J. Membr. Sci..

[26] Zhu L., Peng X., Shang S., Kwasny M.T., Zimudzi T.J., Yu X., Saikia N., Pan J., Liu Z.-K., Tew G.N. (2019). High Performance Anion Exchange Membrane Fuel Cells Enabled by Fluoropoly(olefin) Membranes. Adv. Funct. Mater..

[27] Robertson N.J., Kostalik I.H.A., Clark T.J., Mutolo P.F., Abruña H.D., Coates G.W. (2010). Tunable High Performance Cross-Linked Alkaline Anion Exchange Membranes for Fuel Cell Applications. J. Am. Chem. Soc..

[28] You W., Padgett E., MacMillan S.N., Muller D.A., Coates G.W. (2019). Highly conductive and chemically stable alkaline anion exchange membranes via ROMP of *trans* -cyclooctene derivatives. Proc. Natl. Acad. Sci. USA.

[29] Mandal M., Huang G., Hassan N.U., Mustain W.E., Kohl P.A. (2020). Poly(norbornene) anion conductive membranes: Homopolymer, block copolymer and random copolymer properties and performance. J. Mater. Chem. A.

[30] Gao X., Yu H., Qin B., Jia J., Hao J., Xie F., Shao Z.-G. (2019). Enhanced water transport in AEMs based on poly(styrene–ethylene–butylene–styrene) triblock copolymer for high fuel cell performance. Polym. Chem..

[31] Yu N., Dong J., Li H., Wang T., Yang J. (2021). Improving the performance of quaternized SEBS based anion exchange membranes by adjusting the functional group and side chain structure. Eur. Polym. J..

[32] Lin B., Xu F., Su Y., Zhu Z., Ren Y., Ding J., Yuan N. (2019). Facile Preparation of Anion-Exchange Membrane Based on Polystyrene-*b*-polybutadiene-*b*-polystyrene for the Application of Alkaline Fuel Cells. Ind. Eng. Chem. Res..

[33] Liu L., Li D., Xing Y., Li N. (2018). Mid-block quaternized polystyrene-b-polybutadiene-b-polystyrene triblock copolymers as anion exchange membranes. J. Membr. Sci..

[34] Faraj M., Elia E., Boccia M., Filpi A., Pucci A., Ciardelli F. (2011). New anion conducting membranes based on functionalized styrene-butadiene-styrene triblock copolymer for fuel cells applications. J. Polym. Sci. Part A Polym. Chem..

[35] Jeon J.Y., Park S., Han J., Maurya S., Mohanty A.D., Tian D., Saikia N., Hickner M.A., Ryu C.Y., Tuckerman M.E. (2019). Synthesis of Aromatic Anion Exchange Membranes by Friedel–Crafts Bromoalkylation and Cross-Linking of Polystyrene Block Copolymers. Macromolecules.

[36] Shi Y., Zhao Z., Liu W., Zhang C. (2020). Physically Self-Cross-Linked SEBS Anion Exchange Membranes. Energy Fuels.

[37] Wang Z., Li Z., Chen N., Lu C., Wang F., Zhu H. (2018). Crosslinked poly (2,6-dimethyl-1,4-phenylene oxide) polyelectrolyte enhanced with poly (styrene-b-(ethylene-co-butylene)-b-styrene) for anion exchange membrane applications. J. Membr. Sci..

[38] Filpi A., Boccia M., Pucci A., Ciardelli F. (2013). Modulation of the electrochemical properties of SBS-based anionic membranes by the amine molecular structure. E-Polymers.

[39] Tsai T.-H., Ertem S.P., Maes A.M., Seifert S., Herring A.M., Coughlin E.B. (2015). Thermally Cross-Linked Anion Exchange Membranes from Solvent Processable Isoprene Containing Ionomers. Macromolecules.

[40] Kang Z., Pak M., Bender G. (2021). Introducing a novel technique for measuring hydrogen crossover in membrane-based electrochemical cells. Int. J. Hydrogen Energy.

[41] Faraj M., Boccia M., Miller H.A., Martini F., Borsacchi S., Geppi M., Pucci A. (2012). New LDPE based anion-exchange membranes for alkaline solid polymeric electrolyte water electrolysis. Int. J. Hydrogen Energy.

[42] Sherazi T.A., Sohn J.Y., Lee Y.M., Guiver M.D. (2013). Polyethylene-based radiation grafted anion-exchange membranes for alkaline fuel cells. J. Membr. Sci..

[43] Song J.-M., Lee S.-Y., Woo H.-S., Sohn J.-Y., Shin J. (2014). Thermal behavior of poly(vinylbenzyl chloride)-grafted poly(ethylene-*co* -tetrafluoroethylene) films. J. Polym. Sci. Part B Polym. Phys..

[44] Li Y., Jackson A.C., Beyer F.L., Knauss D.M. (2014). Poly(2,6-dimethyl-1,4-phenylene oxide) Blended with Poly(vinylbenzyl chloride)-*b*-polystyrene for the Formation of Anion Exchange Membranes. Macromolecules.

